# Genome-Wide Analysis of the ERF Family and Identification of Potential Genes Involved in Fruit Ripening in Octoploid Strawberry

**DOI:** 10.3390/ijms231810550

**Published:** 2022-09-11

**Authors:** Yunting Zhang, Chenhui Guo, Meiyi Deng, Shanlin Li, Yingying Chen, Xianjie Gu, Guohao Tang, Yuanxiu Lin, Yan Wang, Wen He, Mengyao Li, Yong Zhang, Ya Luo, Xiaorong Wang, Qing Chen, Haoru Tang

**Affiliations:** 1 College of Horticulture, Sichuan Agricultural University, Chengdu 611130, China; 2 Institute of Pomology and Olericulture, Sichuan Agricultural University, Chengdu 611130, China; 3 Mianyang Academy of Agricultural Sciences, Mianyang 621000, China

**Keywords:** strawberry, ethylene response factors, expression profile, fruit ripening

## Abstract

Ethylene response factors (ERFs) belonging to the APETALA2/ERF superfamily acted at the end of the ethylene signaling pathway, and they were found to play important roles in plant growth and development. However, the information of ERF genes in strawberry and their involvement in fruit ripening have been limited. Here, a total of 235 ERF members were identified from 426 AP2/ERF genes at octoploid strawberry genome level and classified into six subgroups according to their sequence characteristics and phylogenetic relationship. Conserved motif and gene structure analysis supported the evolutionary conservation of FaERFs. Syntenic analysis showed that four types of duplication events occurred during the expansion of FaERF gene family. Of these, WGD/segmental duplication played a major role. Transcriptomic data of *FaERF* genes during fruit ripening and in response to abscisic acid screened one activator (*FaERF316*) and one repressor (*FaERF118*) that were involved in fruit ripening. Transcriptional regulation analysis showed some transcription factors related to ripening such as ABI4, TCP15, and GLK1 could bind to *FaERF316* or *FaERF118* promoters, while protein–protein interaction analysis displayed some proteins associated with plant growth and development could interact with FaERF118 or FaERF316. These results suggested that *FaERF118* and *FaERF316* were potential genes to regulate strawberry ripening. In summary, the present study provides the comprehensive and systematic information on FaERF family evolution and gains insights into FaERF’s potential regulatory mechanism in strawberry ripening.

## 1. Introduction

Transcription factors (TFs) play an important role throughout the entire life cycle of plants. They are usually defined as a kind of specific protein that directly binds with the sequence-specific DNA on cis-acting regulatory elements (CAREs) in the promoter to active or repress transcription of the target genes. It is estimated that Arabidopsis dedicates over 5% of its genome to code for more than 1500 transcription factors, which were initially classified into over 35 TF families [1]. The currently available data show that 58 TF families are found in plants [2]. Among the identified TF multigene families, AP2/ERF, APETALA2/Ethylene Response Factor, is one of the largest superfamilies [1]. It has been reviewed that the AP2/ERF transcription factors play an important role in the transcriptional regulation of multiple biological processes related to plant growth and development. Moreover, they can integrate various internal and external stimuli signals in the biotic and abiotic stress responses [3,4].

AP2/ERF transcription factors are defined by the AP2/ERF domain of around 60 amino acids that directly interact with cis-acting elements in the promoter of the target genes [5,6,7]. According to the number of AP2 domain and sequence similarities, AP2/ERF superfamily has been divided into three major families, namely the AP2 (APETALA2), ERF, and RAV (related to abscisic acid insensitive3/Viviparous1, ABI3/VPI), as well as soloists (few unclassified factors). The AP2 family is characterized by two AP2/ERF domains, whereas the ERF family contains one AP2/ERF domain, and the RAV family is defined by a B3 domain, in addition to a single AP2/ERF domain. The ERF family is further classified into the ERF and the CBF/DREB (C-repeat Binding Factor/Dehydration Responsive Element-Binding) subfamilies based on the difference of 14th and 19th amino acids in the AP2 domain, which is respectively divided into A1–A6 and B1–B6 subgroups [8,9]. The DREB proteins predominantly participate in abiotic stress response by recognizing an A/GCCGAC element [10], while the ERF proteins are mainly involved in ethylene, pathogens, and wounding response by specifically binding to an AGCCGCC element (GCC box) [11].

ERF proteins are the downstream elements of the ethylene signaling pathway and have received much attention on climacteric fruit which need a burst of ethylene production to initiate ripening [12], while ABA signaling pathway plays a core role in non-climacteric fruit ripening [13]. In recent years, ethylene signaling has been found to participate in regulating non-climacteric fruit ripening as well by interacting the ABA signaling, as elucidated by AREB/ABF-mediated ACS/ACO expression and ERF-mediated NCED expression [14,15]. A growing body of evidence has proposed that ERF proteins can affect different aspects (color, aroma, and flavor) of fruit ripening in non-climacteric fruits [16]. Xie et al. (2014) [17] systematically analyzed 126 ERF genes in citrus and found that CitERF60 may be associated with fruit chlorophyll degradation. In longan, the DlERF1 interacts with histone deacetylase HD2 to regulate fruit senescence [18].

Strawberry is a model horticultural crop for investigating the mechanism of non-climacteric fruit ripening. Research on its ripening during the past years has focused on the ABA signaling pathway. However, with more and more studies suggesting that ethylene may play a regulatory role in strawberry fruit development and ripening, the regulatory mechanisms involved have not yet been revealed. Here, we intended to systematically characterize the ethylene response factor (ERF) family in octoploid strawberry at genome level and conduct a comprehensive analysis on phylogenetic construction, chromosomal distribution, conserved motif, and gene structure. Moreover, the expression patterns of the *ERF* members in strawberry different ripening stages and ABA treatment were analyzed. The results provided a basis for further investigating the roles of ERF transcription factors and ethylene in strawberry or other non-climacteric fruit ripening.

## 2. Results

### 2.1. Identification and Characterization of FaERFs

**Figure 1:**
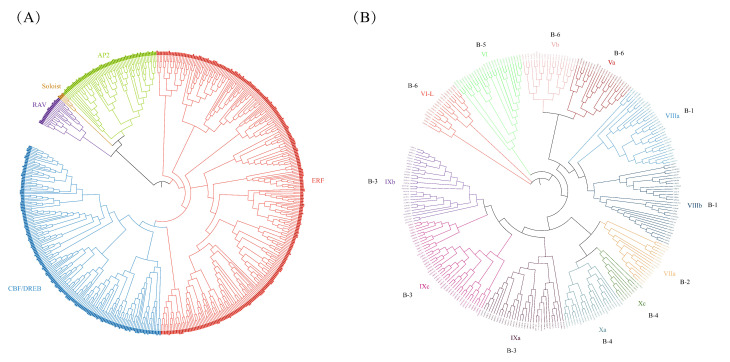
Phylogenetic analysis of the AP2/ERF superfamily (**A**) and ERF subfamily (**B**) between Arabidopsis and strawberry. At, *Arabidopsis thaliana*; Fa, *Fragaria × ananassa*.

Through HMM analysis and local BLAST search, a total of 426 AP2/ERF genes were identified in the octoploid strawberry genome. The phylogenetic tree was obtained on a basis of the alignment of AP2/ERF proteins from *Fragaria × ananassa* and *Arabidopsis* (Figure 1A). According to the classification of AP2/ERF in *Arabidopsis*, the phylogenetic tree clearly divided the AP2/ERF superfamily from *Fragaria × ananassa* into five subfamilies: AP2 (51 members), RAV (15 members), ERF (235 members), and CBF/DREB (122 members), as well as Soloists (3 members). Then, the ERF subfamily was further classified into six subgroups, named B-1 to B-6, which contained 47, 12, 83, 24, 18, and 51 members, respectively (Figure 1B). The identified FaERF proteins ranged from 93 to 707 amino acids in length, with theoretical pI varying from 4.54 to 10.73 and molecular weights ranging from 10,254.54 to 79,412.32 Da. Subcellular localization analysis predicted that the majority of FaERFs were localized to the nucleus, whereas other members were localized to the chloroplast, peroxisome, mitochondrion, or cytoplasm (Table S1).

### 2.2. Gene Structure and Conserved Motif of FaERFs

**Figure 2:**
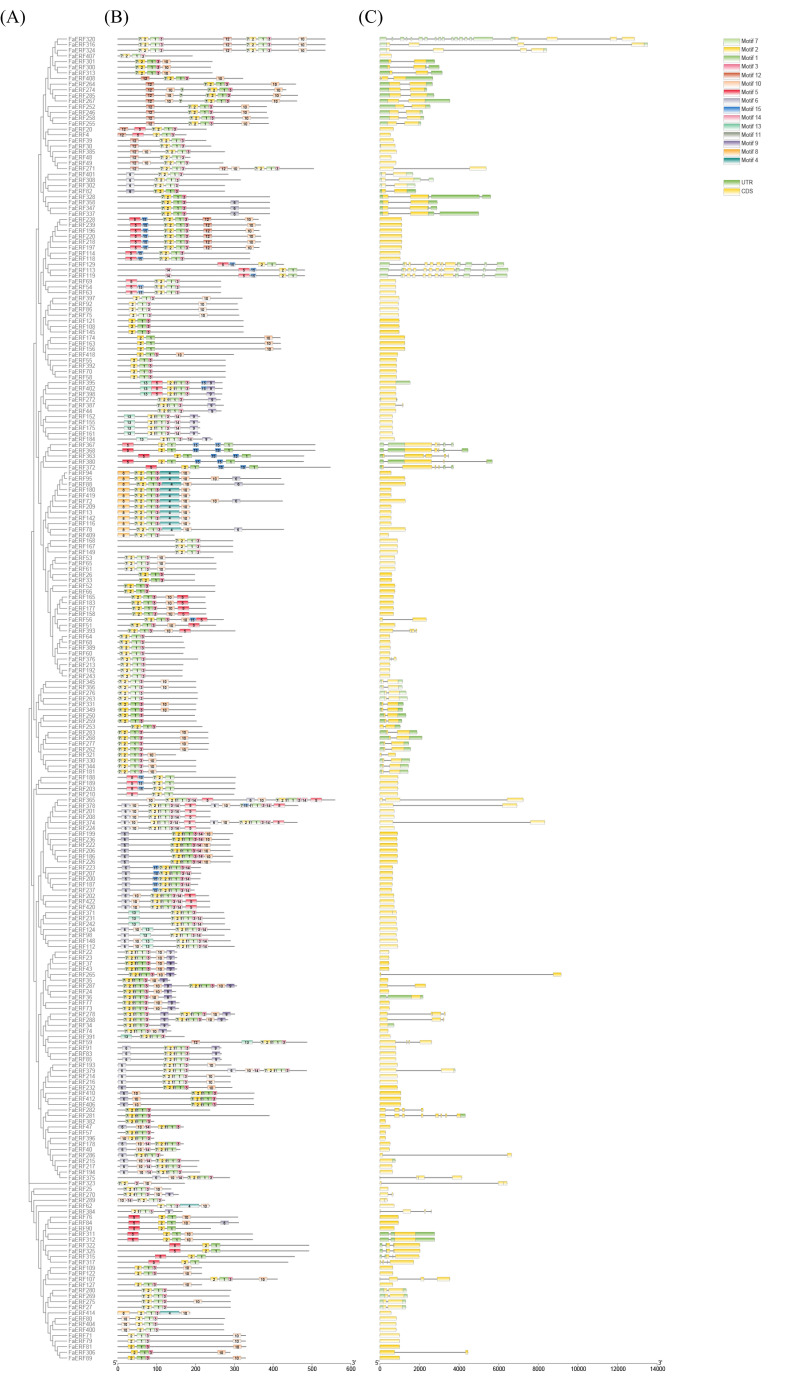
Phylogenetic relationship, conserved protein motif, and gene structure analysis of FaERFs from strawberry. (**A**) The phylogenetic tree of the FaERF gene family. (**B**) Distribution and composition of motifs of the FaERF gene family. (**C**) Exon–intron structure of the FaERF gene family.

To better understand the gene structural characteristics of the FaERF subfamily, the intron/exon arrangement of each *FaERF* gene was analyzed. More than half of the *FaERFs* were intronless, and other *FaERF* coding regions were interrupted by introns of varying sizes. Furthermore, the conserved motifs of FaERF subfamily were analyzed using MEME. A total of 15 motifs were predicted and named as motifs 1 to 15. The motifs 1 and 2 were distributed in almost all FaERFs, while other motifs were distributed among various subgroups. Moreover, conserved motif and gene structure analysis suggested that the motif composition and distribution of FaERF proteins and their gene structures were conserved within each subfamily, but diverged widely among subfamilies (Figure 2).

### 2.3. Chromosomal Location and Synteny Analysis of FaERFs

**Figure 3:**
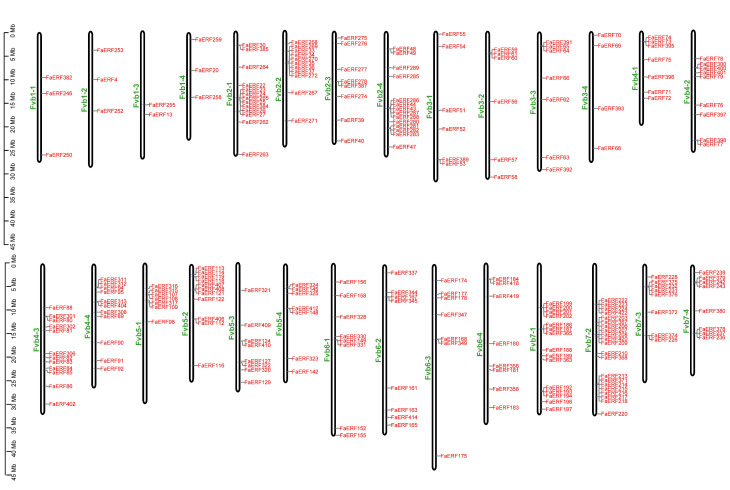
Chromosome distribution of *FaERF* genes in strawberry.

**Figure 4:**
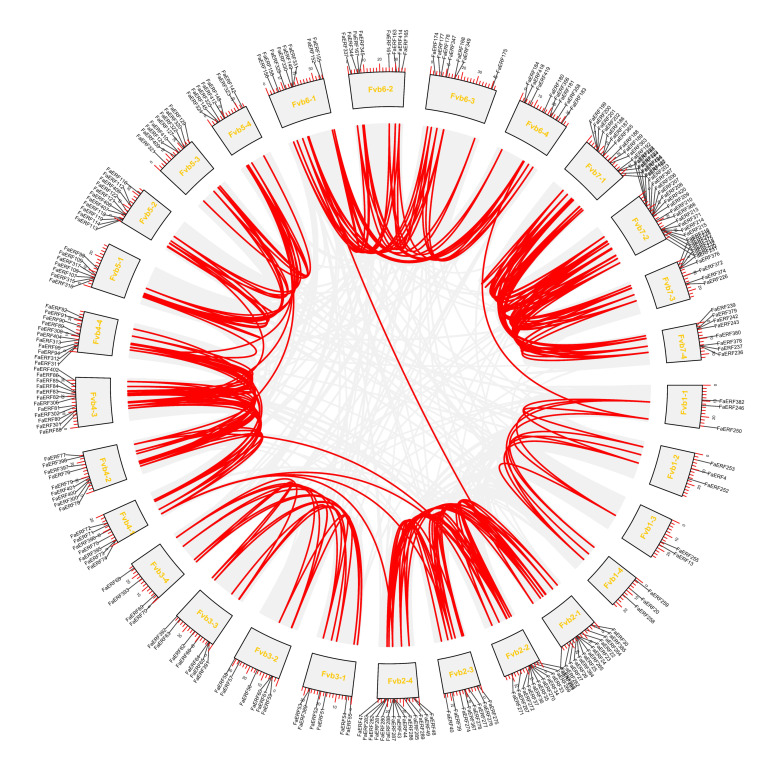
Synteny analysis of *FaERF* genes in strawberry.

To better understand the genomic distribution of *FaERF* genes, their positions on each chromosome were investigated. As shown in Figure 3, 235 *FaERF* members were distributed unevenly on 28 chromosomes. Chromosome 7-2 had the largest number (21, 8.94%) of *FaERF* genes, containing 13 of B-3 subgroup members, 4 of B-5 subgroup members, 2 of B-6 subgroup members, 2 of B-1 subgroup members, while Chromosome 1-3 only had two members. Chromosomes 7-1, 2-4, 2-1 had 6.38% (15/235), 5.96% (14/235), and 5.53% (13/235) *FaERF* genes, respectively. The remaining members were located on the rest of the chromosomes. The synteny analysis showed that a total of 272 pairs of *FaERF* genes were identified as collinear pairs in strawberry (Figure 4, Table S2). Subsequently, the duplication events of *FaERFs* were characterized using MCScanX package. Four types of duplication events including proximal, dispersed, whole genome duplication (WGD) or segmental (WGD/segmental), and tandem were found. Among them, there were 205 cases of WGD/segmental, occupying about 87.7% of total duplication events, followed by dispersed duplication with 23 genes, accounting for 9.8%, followed by proximal duplication with 5 genes (*FaERF311*, *FaERF312*, *FaERF282*, *FaERF385* and *FaERF129*), explaining 2.1%. Only *FaERF270* was duplicated from tandem duplication, accounting for 0.4%. These findings indicated that the expansion of *FaERFs* was guided by WGD/segmental (Figure 4, Table S1).

### 2.4. Expression Profiles Analysis of FaERF Genes

**Figure 5:**
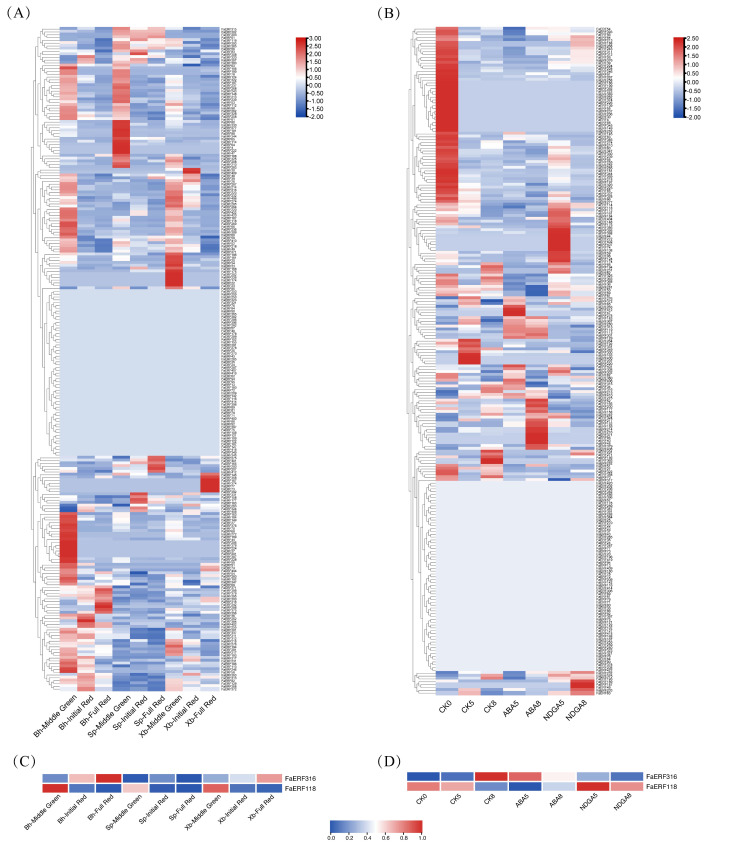
Expression profiles of *FaERF* genes in strawberry fruit. (**A**) Expression profiles of *FaERFs* during fruit development and ripening of three strawberry cultivars. (**B**) Expression profiles of *FaERFs* in ABA-treated fruits of the ‘Toyonoka’ cultivar. (**C**) Expression profiles of *FaERF118* and *FaERF316* during fruit development and ripening of three strawberry cultivars. (**D**) Expression profiles of *FaERF118* and *FaERF316* in ABA-treated fruits of the ‘Toyonoka’ cultivar. Bh, ‘Benihoppe’; Sp, ‘Snow Princess’; Xb, ‘Xiaobai’; CK0, fruits injected with distilled water on day 0; CK5, fruits injected with distilled water on day 5; CK8, fruits injected with distilled water on day 8; ABA5, fruits injected with ABA on day 5; ABA8, fruits injected with ABA on day 8; NDGA5, fruits injected with NDGA on day 5; NDGA8, fruits injected with ABA on day 8.

**Figure 6:**
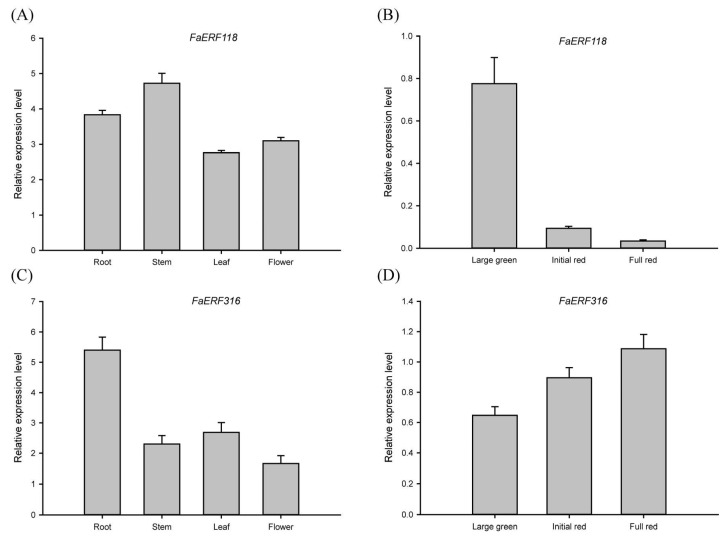
Expression patterns of *FaERF118* and *FaERF316* in the ‘Benihoppe’ strawberry. (**A**,**C**) Relative expression levels of *FaERF118* and *FaERF316* in different tissues. (**B**,**D**) Relative expression levels of *FaERF118* and *FaERF316* during fruit development.

To identify the FaERFs involved in fruit ripening, the expression patterns of *FaERFs* during fruit development and in ABA-treated fruits were examined. As shown in Figure 5A,B, some of *FaERFs* were not expressed in fruits, while other members were expressed at varying levels during fruit development and ripening. Many *ERF* genes showed a different change trend in different cultivars, indicating that ERFs could regulate the cultivar-specific traits. For instance, *FaERF51* was upregulated during ‘snow princess’ ripening, whereas it almost had no change in other cultivars and maintained at a very low level. Interestingly, we found that the transcript abundance of *FaERF316* was increasing during fruit development and ripening of different cultivars. Moreover, it was induced by ABA and inhibited by ABA biosynthesis blocker nordihydroguaiaretic acid (NDGA). However, the expression level of *FaERF118* had the opposite change trend (Figure 5C,D). Furthermore, we quantified the expression of *FaERF118* and *FaERF316* in three different stages of ‘Benihoppe’ fruit by qRT-PCR analysis to validate the accuracy of the transcriptome profiles. The results showed that *FaERF118* decreased when fruit turned red, while *FaERF316* increased and peaked at full red stage, which was consistent with the transcriptome data (Figure 6B,D). Therefore, we speculated that FaERF118 and FaERF316 were involved in the fruit ripening process. In addition, *FaERF118* and *FaERF316* demonstrated tissue-specific expression patterns. *FaERF118* had the highest expression level in stem, followed by root, flower, and leaf, while *FaERF316* displayed the highest transcript abundance in root, followed by leaf, stem, and flower (Figure 6A,C). These findings suggested that the *FaERF118* and *FaERF316* may play a broad role in strawberry growth and development.

### 2.5. Regulatory Mechanism Analysis in the Promoter Regions of FaERF118 and FaERF316

**Figure 7:**
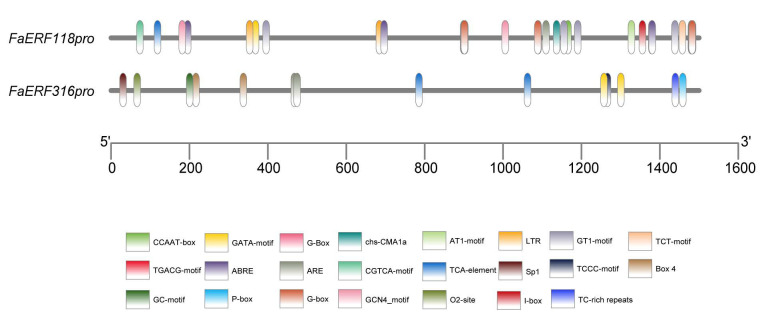
Cis-acting regulatory elements in the *FaERF118* and *FaERF316* promoters.

The upstream 1500 bp fragments from the start codon in *FaERF118* and *FaERF316* genes were respectively isolated to predict the cis-acting regulatory elements. In addition to multiple core promoter elements, TATA-box and enhancers CAAT-box, both promoters contained several light responsive elements such as GATA-motif, G-box, chs-CMA1a, GT1-motif, AT1-motif, TCT-motif, Sp1, TCCC-motif, and Box 4. They also included hormone-related and stress-related cis-acting elements. Notably, the promoter of *FaERF118* had many ABRE cis-acting elements, indicating it was sensitive to ABA signaling (Figure 7). To further explore the transcriptional regulation of *FaERF118* and *FaERF316*, we analyzed potential TFs that can bind to these two *ERF* promoters. The results showed 15 kinds of TFs were predicted to bind to the *FaERF118* promoter, while 9 kinds of TFs were found to bind to the *FaERF316* promoter (Table 1). The biological functions of these TFs were related to stress response, plant development, and hormone response. Of these, it was worth noting that the TFs predicted in *FaERF118* promoters such as TCP15 were annotated to act as a repressor of anthocyanin accumulation [19] and GLK1 was annotated to be the activator of chlorophyll biosynthesis [20]. ABI4 predicted in *FaERF316* promoter was a critical downstream component of ABA signaling in plant [21,22], while bZIP53 could affect plant sugar accumulation [23,24]. 

### 2.6. Protein–Protein Network Analysis of FaERF118 and FaERF316

**Figure 8:**
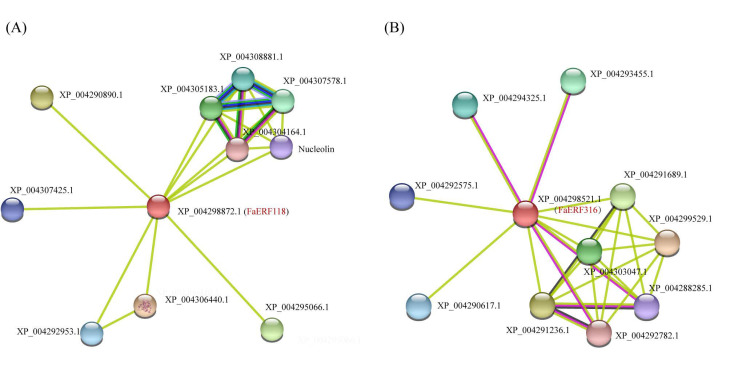
Interaction network analysis of FaERF118 (**A**) and FaERF316 (**B**) identified in *Fragaria × ananassa* based on the respective homologous in *Fragaria × vesca*. XP_004298872.1, Ethylene-responsive transcription factor crf2-like (CRF2); XP_004306440.1, ETHYLENE INSENSITIVE 3-like (EIN3); XP_004290890.1, NAC domain-containing protein 2-like (NAC2); XP_004295066.1, Putative calmodulin-like protein 2 (CML2); XP_004305183.1, XP_004307578.1, XP_004308881.1, Delta-1-pyrroline-5-carboxylate synthase-like (P5CS); XP_004292953.1, Transcription factor E2FC-like (E2FC); XP_004307425.1, Ethylene-responsive transcription factor RAP2-7-like (RAP2-7); Nucleolin, Uncharacterized protein LOC101311800; XP_004304164.1, Proline dehydrogenase 2 (ProDH2); XP_004298521.1, Ethylene-responsive transcription factor erf113-like (ERF113-like); XP_004299529.1 Probable ADP-ribosylation factor GTPase-activating protein AGD11-like (AGD11-like); XP_004291236.1, Zinc finger protein 622-like (ZFP622); XP_004291689.1, NAC domain-containing protein 89-like (NAC 89); XP_004303047.1, Uncharacterized protein LOC101312168; XP_004293455.1, Scarecrow-like transcription factor PAT1-like (PAT1); XP_004294325.1, Chitin-inducible gibberellin-responsive protein 1-like (CIGR1); XP_004290617.1, XP_004292575.1, Non-specific lipid-transfer protein-like protein At2g13820-like; XP_004288285.1, Transcription factor RAX3-like (RAX3); XP_004292782.1, Uncharacterized protein LOC101303879.

To predict the molecular interactions between FaERF118 or FaERF316 and other proteins, an interaction network was constructed based on the orthologous gene of diploid strawberry (*Fragaria × vesca*). FvCRF2 (homologous to FaERF118), a cytokinin response factor, was related to plant growth and development. It was predicted that FvCRF2 can interact with the factors (EIN3, ERF RAP2-7) in ethylene signaling pathway. Moreover, FvCRF2 was computed to associate with NAC2, CML2, E2FC, P5CS, and ProDH2 proteins, which played roles in plant development and stress response. Accordingly, FvERF113 (homologous to FaERF316) was predicted to interact with AGD11, ZFP622, NAC89, PAT1, CIGR1, and RAX3 proteins, which were also involved in plant development and stress response (Figure 8).

## 3. Discussion

Ethylene-responsive factors (ERFs) belong to the AP2/ERF large superfamily and act at the end of the ethylene signaling pathway to regulate the expression of ethylene-responsive genes. In line with the function of ethylene in multiple biological processes, ERF proteins have been demonstrated to play a pivotal role in plant growth, maturation, senescence, and stress response [16,25,26]. It has been well documented that ERFs were essential in initiating and orchestrating the ripening of climacteric fruit that display a respiration and ethylene burst at the onset of ripening [27,28], whereas the role of ERFs in non-climacteric fruits have not attracted much attention, since non-climacteric fruits have no significant change in respiration and ethylene burst during ripening. Recently, increasing evidence have shown that ERFs also participate in various aspects of the fruit ripening process in non-climacteric fruits, which can be regulated by ethylene or other factors [15,16]. Thus, the identification and characterization of ERFs, along with screening candidate ERF genes involving the non-climacteric fruit ripening, is a significant investigation.

Strawberry is a typical non-climacteric fruit. Edger et al. (2019) [29] developed the chromosome-scale *F. × ananassa* ‘Camarosa’ reference genome, which can serve as a powerful genetic resource to unravel the complexity of octoploid strawberry genome for gene-trait association studies, including identification of TFs regulating fruit ripening in strawberry breeding programs. In the present study, a total of 235 FaERF members were identified from the octoploid strawberry genome, which is far more than the number existing in the genomes of diploid woodland strawberry [30], Chinese jujube [31], grape [32], pomegranate [33], apple [34], pear [35], citrus [17] and other fruit trees. This finding indicated that FaERFs largely expanded in the octoploid strawberry. The published data have demonstrated that ERF subfamily has the most members in AP2/ERF superfamily [33]. Phylogenetic trees of ERFs derived from strawberry and Arabidopsis showed that most of the clusters in the ERF family contained both genes from these two species, indicating these ERF genes have established prior to the evolutionary divergence of species. Moreover, FaERF genes in the same cluster share the similar conserved motifs and exon-intron structures. The previous studies have suggested that genes in the same subgroup decide the similar gene function and could predict ERF function according to the known function of ERFs in Arabidopsis [35,36]. However, the Arabidopsis is a plant without fleshy fruit. It is relatively limited to perform functional characterization of genes involving strawberry fruit ripening using the AtERFs as the queries in the phylogenetic tree.

Gene duplication events such as tandem duplication, WGD, and segmental duplication occurring during genome evolution are the main sources that give rise to gene expansion, and they have remarkable roles in plant growth, development, and adaptability to various stresses. Our result showed that the expansion of *FaERFs* was mainly due to WGD/segmental duplication, which was consistent with our previous studies in *MAPK* [37], *UBC* [38], *GMP* [39], and *GST* [40] gene families of the cultivated strawberry. In addition, we found that the number of *FaERFs* in the B-3 group was significantly greater than other groups by gene duplication, implying that B-3 of FaERFs had significant expansion through the evolution of cultivated strawberries. This finding suggests the B-3 of FaERFs is of importance for plant to adapt significant environmental changes.

Despite the potential role of FaERFs in fruit development and maturation, only a very few FaERF members have been identified to regulate non-climacteric fruit ripening. Zhang and Li (2018) [31] eventually screened one activator (*ZjERF54*) and two repressors (*ZjERF25* and *ZjERF36*) associated with jujube fruit ripening by gene expression analysis during fruit ripening and in response to ethylene. *VviERF6L1* was considered to participate in grape ripening, since its transcript abundance had the largest changes in the skin and clustered with genes involved in ethylene, senescence, and fruit flavor production [41]. It has been well demonstrated that ABA signaling has the core role in mediating non-climacteric fruit ripening. Interestingly, the ABA signaling can merge ethylene signaling by ERFs [15]. These findings suggest that ERFs may involve in ABA-mediated non-climacteric fruit ripening. Herein, we analyzed the expression profile of *FaERFs* during fruit ripening process and in ABA-treated fruit. The results showed *FaERF316* was up-regulated during ripening and induced by ABA treatment, indicating *FaERF316* was positively correlated with strawberry fruit maturation. By contrast, *FaERF118* might be negatively associated with strawberry fruit ripening. In agreement with this argument, the output of transcriptional regulation prediction showed that the ABI4 and bZIP53 could bind to the promoter of *FaERF316*. The homolog ABI4 in strawberry was demonstrated to act a positive regulator for fruit ripening by affecting anthocyanin accumulation, firmness, and ABA content, and the expression of *ABI4* could be induced by ABA, sucrose, and glucose [22], while the homolog bZIP53 in strawberry was found to regulate sugar accumulation [24,42]. Conversely, TCP15 and GLK1 were predicted to bind to the promoter of *FaERF118*. The TCP15 can inhibit the anthocyanin accumulation [19], which is an important indicator of ripeness in strawberries. GLK1, the known nuclear transcript factor directly required for the transcription of genes encoding chloroplast proteins for chloroplast development [20]. Moreover, the promotor of *FaERF118* included several ABA-responsive cis-acting elements, and FaERF118 and EIN3 had the protein–protein interaction. These results suggested that FaERF316 and FaERF118 probably regulated strawberry ripening through the interplay between ABA and ethylene signaling.

## 4. Materials and Methods

### 4.1. Plant Materials, RNA Extraction, cDNA Synthesis

To analyze the tissue-specific expression profile of the selected *FaERF* genes, different tissues of root, stem, leaf, flower, and fruit at three developmental stages (large green, initial red; full red) were collected from a local orchard located in Shuangliu County, Sichuan Province, south-west China. These materials were quickly frozen in liquid nitrogen and stored at −80 °C for further experiments. The Total RNA was extracted from all samples via the improved CTAB method described by Chen et al. (2012) [43]. The first strand of cDNA was synthesized using the PrimeScriptTM RT reagent Kit with gDNA Eraser (Takara, Japan).

### 4.2. Identification of FaERF Genes in Strawberry

The genome sequence and annotation information of *Fragaria × ananassa* were downloaded from the Genome Database for Rosaceae (GDR) (https://www.rosaceae.org/species/fragaria_x_ananassa/genome_v1.0.a1, accessed on 3 July 2020). Both local Blast and HMMER searches were used to identify the AP2/ERF sequences in strawberry. The Arabidopsis AP2/ERF family consisting of 18 AP2, 6 RAV, 57 DREB, 65 ERF genes, and 1 soloist were retrieved from The Arabidopsis Information Resource (TAIR) (https://www.arabidopsis.org/index.jsp, accessed on 27 July 2020) database to conduct a local Blast search. Meanwhile, The AP2-specific hidden Markov Model (HMM) profile (PF00847) downloaded from the Pfam protein families database (https://pfam.xfam.org, accessed on 3 August 2020) was used as the query to search for AP2/ERFs in the HMMER 3.0 program with a defined e-value threshold < 1e-5. Then, the putative sequences were obtained by integrating both results above. After that, all candidate AP2/ERFs were further filtered based on their conserved domains by SMART (http://smart.embl-heidelberg.de/, accessed on 3 August 2020) and Conserved Domain Database (CDD) (https://www.ncbi.nlm.nih.gov/Structure/cdd/wrpsb.cgi, accessed on 12 August 2020). A phylogenetic tree of AP2/ERFs in strawberry and Arabidopsis was generated using the neighbor-joining method of MEGA-X with the 1000 bootstrap replicates and Poisson model, after a multiple sequence alignment of all AP2/ERF protein sequences encoding the conserved AP2 domain was constructed with MUSCLE. Subsequently, the putative FaERF subfamilies were characterized and classified into different groups.

### 4.3. Sequence Analysis of FaERF Genes

The basic physicochemical properties of amino acid length, theoretical pI, molecular weight, grand average of hydropathicity (GRAVY), aliphatic index, and instability index of FaERF protein were evaluated by ExPASy-ProtParam online website (http://web.expasy.org/protparam/, accessed on 20 July 2021); the signal peptide was predicted by SignalP 4.1 Server (http://www.cbs.dtu.dk/services/SignalP/, accessed on 16 September 2021); the transmembrane structure and subcellular location were analyzed by TMHMM Server v. 2.0 (http://www.cbs.dtu.dk/services/TMHMM/, accessed on 23 July 2021) and ProtComp v.9.0 (http://linux1.softberry.com/berry.phtml?topic=protcomppl&group=programs&subgroup=proloc, accessed on 24 July 2021), respectively. The cis-regulatory elements in the 1500 bp upstream region of the candidate gene promoters were identified using the PlantCARE online tool (http://bioinformatics.psb.ugent.be/webtools/plantcare/html/, accessed on 23 June 2022). The transcription factor binding sites in candidate gene promoters were predicted using the PlantTFDB database (http://plantregmap.gao-lab.org/binding_site_prediction.php, accessed on 24 June 2022). The interaction networks of the candidate proteins were analyzed by STRING (https://string-db.org/, accessed on 24 June 2022).

### 4.4. Gene Structure and Conserved Motif Analysis of FaERF Genes

The exon–intron structure of *FaERF* genes was analyzed based on the comparison of on the full-length genome sequences and the corresponding coding sequences, and graphically displayed with Gene Structure Display Server program (GSDS v.2.0, http://gsds.cbi.pku.edu.cn, accessed on 27 July 2021). Conserved motifs in FaERFs were identified using the Multiple Em for Motif Elicitation (MEME) web server (http://meme-suite.org/tools/meme, accessed on 4 August 2021) using default parameters.

### 4.5. Chromosomal Mapping and Synteny Analyses of FaERF Genes

The information on the location of *FaERFs* on a chromosome was retrieved from the annotated file of the *Fragaria × ananassa* Camarosa Genome Assembly v1.0 & Annotation v1.0.a1 and graphically mapped with Tbtools. The Multiple Collinearity Scan toolkit (MCScanX) was used to determine *FaERF* gene synteny and collinearity, and the synteny plot was visualized with Tbtools [44,45]. Tandem duplications were characterized as two or more homologous genes occurring in a chromosome region (distance < 200 kb).

### 4.6. Expression Profile Analysis of FaERF Genes

All quantitative real-time PCRs were conducted using SYBR Premix (Takara, Maebashi, Japan) on the CFX96 real-time PCR system (Bio-Rad, Hercules, CA, USA). The calculation of the relative gene expression levels followed the 2^−ΔΔCT^ method, and the *Actin* was used as the internal control to normalize the expression of target genes. Primers used for qRT-PCR are listed in Table S3. The RNAseq-based expression patterns of *FaERF* genes in strawberry were retrieved from the online transcriptomic data that had been submitted to the NCBI database (PRJNA552213; PRJNA338879). The samples used for PRJNA552213 transcriptomes were obtained from three fruit development and ripening stages (middle green, initial red, and full red) of three octoploid cultivated strawberry varieties (‘Benihoppe’ with red fruit skin and flesh; ‘Xiaobai’, the white-flesh mutant of ‘Benihoppe’; ‘Snow Princess’ with white fruit skin and flesh) [46]. The samples used for PRJNA338879 transcriptomes were obtained after pharmacological treatment. Two-week old fruits post-anthesis were injected with 100 μL of ABA (1 μM), the ABA biosynthesis blocker nordihydroguaiaretic acid (NDGA, 100 μM), or distilled water (used as a control). Samples were harvested on day 5 and 8, and correspondingly denoted as CK5, CK8, ABA5, ABA8, NDGA5, and NDGA8. Additionally, receptacles were immediately harvested after fruits were injected with distilled water and the sample was named CK0 [47].

## 5. Conclusions

In this study, a total of 235 *FaERF* genes were identified in octoploid strawberry genome after a comprehensive and systematic analysis. Their phylogenetic relationship, gene structure, conserved motifs/domain distribution, chromosome location, and synteny analysis contributed to classify these genes and give a better understanding of *FaERF* gene evolution. Expression patterns of *FaERF* genes during fruit ripening and in response to ABA characterized two candidate *FaERF* genes (*FaERF118* and *FaERF316*) associated with strawberry ripening. *FaERF316* might play a positive role in fruit ripening, while *FaERF118* had an opposite effect on this process. In addition, the tissue-specific expression profiles of *FaERF118* and *FaERF316* showed that they expressed in all tissues including root, stem, leaf, flower, and fruit, indicating their broad regulation role in strawberry growth and development. The regulatory mechanism analysis in the promoter regions and protein-protein network analysis of two candidate *FaERF* genes provided deeper insights into their function in strawberry fruit ripening.

## Figures and Tables

**Table 1:** Transcription factors with potential binding sites in promoter regions of *FaERF118* and *FaERF316.*

Promoter	Transcription Factor Gene Family
*FaERF118-pro*	bHLH (BIM1, PIF3), Trihelix (ASIL2), NAC, TCP (TCP19, TCP20, TCP15), C2H2 (ZAT10, TFIIIA), ARF (ARF6, ARF5), MIKC_MADS (AGL22), GRAS (RGA1), CPP (SOL2), BBR-BPC (BPC6, BPC2), CAMTA (CMTA5), bZIP (AREB1, AREB3), DOF (DOF2.4, DOF1.5, DOF 5.1, DOF 1), MYB (MYB50, MYB60), G2-like (GLK1)
*FaERF316-pro*	bZIP (bZIP53, GBF3, bZIP42, bZIP44), Trihelix (ASR3), GRAS (RGA 1), ERF (ERF005, ABI4), BBR-BPC (BPC6, BPC2), MYB (MYB121, MYB50, MYB27, MYB83, MYB84), C2H2 (JKD, TFIIIA) DOF (DOF2.4, DOF 5.1, DOF 5.4), LBD (ASL4, AS2)

Note: the TFs in the bracket were the best hit in Arabidopsis.

## Data Availability

Not applicable.

## Supplementary Materials

The supporting information can be downloaded at: https://www.mdpi.com/article/10.3390/ijms231810550/s1.
